# HuR enhances SARS-CoV-2 non-structural protein translation through the genomic 5′-UTR, by promoting polypyrimidine tract-binding protein binding

**DOI:** 10.1128/jvi.00276-26

**Published:** 2026-04-16

**Authors:** Harsha Raheja, Risabh Sahu, Trinath Ghosh, Santu Paul, Priya Rani, Ashish Aneja, Biju George, Oyahida Khatun, Shashank Tripathi, Saumitra Das

**Affiliations:** 1 Department of Microbiology and Cell Biology, Indian Institute of Science 29120, Bangalore, India; 2 National Institute of Biomedical Genomics 214255, Kalyani, West Bengal, India; 3 Centre for Infectious Disease Research, Indian Institute of Science 29120, Bangalore, India; University of Michigan Medical School, Ann Arbor, Michigan, USA

**Keywords:** 5′-untranslated region, genomic RNA, human antigen R, severe acute respiratory syndrome coronavirus 2, translation

## Abstract

Severe acute respiratory syndrome coronavirus 2 (SARS-CoV-2) viral RNA associates with different RNA-binding host proteins at each stage of its life cycle. We found sequence-dependent binding of one such important protein, human antigen R (HuR), to the SARS-CoV-2 5′-UTR and studied its potential role in the viral life cycle. The knockdown and knockout studies revealed the importance of such binding in viral translation. We identified 5′-UTR mutations in SARS-CoV-2 variants of concern that altered the HuR-binding affinity. Interestingly, HuR enhanced non-structural protein translation through the genomic 5′-UTR by promoting polypyrimidine tract-binding protein binding to the 5′-UTR. HuR knockout increased the sensitivity to remdesivir treatment by decreasing its half-maximal inhibitory concentration. An antisense oligonucleotide (whose binding site overlapped the HuR-binding site) reduced viral RNA levels in wild-type cells but not HuR-knockout cells. Our results indicate that HuR regulates the balance between SARS-CoV-2 structural and non-structural proteins and guides the infection of viral variants, implying that HuR can potentially be explored as an antiviral target.

**IMPORTANCE:**

Viruses interact with various host proteins throughout their life cycle. A key protein is HuR, an RNA-binding protein regulating RNA stability and translation. HuR binds to viral RNAs at the 5′-UTR or 3′-UTR, influencing translation and replication. We identified conserved HuR binding sites in the SARS-CoV-2 5′-UTR across beta coronaviruses. This binding enhances translation initiation from the genomic 5′-UTR, increasing non-structural protein production essential for replication. Additionally, we discovered that another host protein, PTB, is recruited by HuR to the viral 5′-UTR, aiding ribosome loading. This regulation shows that the virus exploits HuR for its benefit. Targeting HuR may help control the SARS-CoV-2 life cycle. HuR knockout increased sensitivity to remdesivir, an antiviral drug. Using an antisense oligonucleotide to block HuR binding effectively reduced viral RNA levels. Our findings highlight HuR’s critical role in viral protein production regulation and its potential as a therapeutic target against SARS-CoV-2.

## INTRODUCTION

Severe acute respiratory syndrome coronavirus 2 (SARS-CoV-2) is a positive-strand RNA virus with a 30 kilobase genome. The viral life cycle begins with attachment of the virus to the angiotensin-converting enzyme 2 (ACE2) receptor. That interaction is followed by receptor-mediated endocytosis, where the virus particles are internalized into endosomes, which release viral RNA into the host cell cytoplasm. Viral RNA undergoes translation to generate a polyprotein that codes for nonstructural proteins. These non-structural proteins initiate replication on the viral 3′-untranslated region (3′-UTR), generating negative-strand genomic RNA. The positive-sense genomic RNA is also used as a template to synthesize negative-sense subgenomic RNAs, which are transcribed to positive-sense subgenomic RNAs coding for viral structural and accessory proteins. The structural proteins thus formed coat the viral positive-strand RNA to form infectious virions, which are released from cells (1).

SARS-CoV-2 RNA and proteins interact with several host proteins to modulate viral pathogenesis. The viral RNA possesses binding sites for cellular RNA-binding proteins (RBPs), which assist the above-mentioned processes at different levels (2, 3). The viral coding region is flanked by UTRs at both the 5′ and 3′ ends, which provide scaffolds for viral and cellular RBPs to bind and regulate translation, replication, or packaging. A dynamic association exists between RBPs and viral RNA during different stages of viral infection. High-throughput screens have identified many RBPs, such as IMP-1, DDX-1, and hnRNPs, that bind to SARS-CoV-2 genomic RNA (4, 5). Here, we studied RBPs predicted to bind to the viral 5′-UTR. Of these, we selected human antigen R (HuR; also known as ELAVL1) for further study.

HuR is an RBP that binds to AU-rich elements in the 3′-UTRs of target mRNAs and stabilizes mRNAs (6, 7). HuR shuttles between the nucleus and cytoplasm and participates in the cellular transport of various RNAs. Different post-translational modifications of HuR have been linked to different functional roles and subcellular localization sites in cells (8). HuR can regulate the replication of hepatitis C virus (9, 10), coxsackievirus B3 (11), and adenovirus (12); stabilize Sindbis virus RNA (13); and modulate human immunodeficiency virus reverse transcription (14). It was also shown to bind the SARS-CoV-2 genome in genome-wide screens (4, 15). We confirmed that HuR can bind the viral 5′-UTR through multiple independent assays and studied its importance in the SARS-CoV-2 life cycle. We examined the potential roles of HuR in regulating the translation of genomic RNA. Furthermore, we blocked HuR-SARS-CoV-2 genome interactions with specific antisense oligonucleotides (ASOs), suggesting that they can be explored as therapeutics for treating COVID-19 disease.

## RESULTS

### HuR regulated the SARS-CoV-2 life cycle

The 5′-UTR of SARS-CoV-2 is 265 nucleotides (nt) long. Enzymatic probing showed that the initial 300 nts of the SARS-CoV-2 genome at the 5′ end form five stem-loop secondary structures that are conserved across all emerging variants (16). The RNA-binding protein database (17) (RBPDB) was used to identify trans-acting host factors that interact with the 5′-UTR of SARS-CoV-2. Several proteins showed 100% relative binding scores, including A2BP1, ZRANB2, sap-49, FUS, PUM2, SRSF9, MBNL1, KHSRP, RBMX, SFRS1, and HuR (see Fig. S1A and B at https://doi.org/10.6084/m9.figshare.31628611). Bioinformatics-based predictions of RBPs with the RBPDB were based on matching linear sequence motifs.

**Fig 1:**
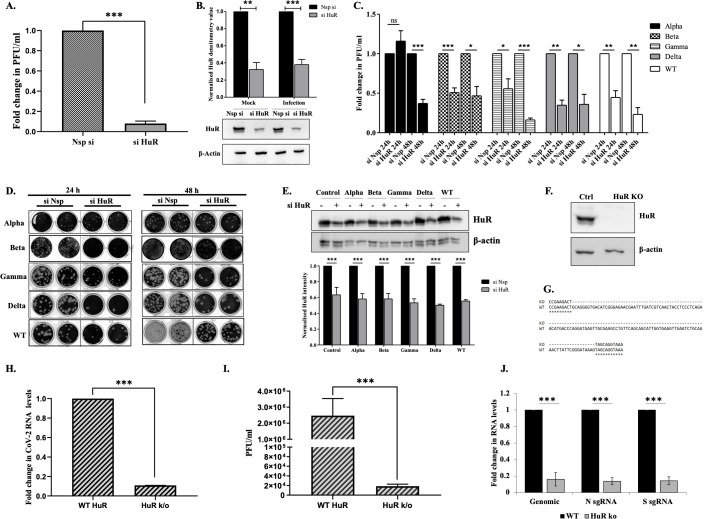
Role of HuR in SARS-CoV-2 lifecycle. (**A**) HEK-293T-ACE2 cells were transfected with either non-specific siRNA (Nsp si) or siRNA targeting HuR (si HuR). Sixteen hours post-transfection, cells were infected with 0.1 MOI of SARS-CoV-2 virus. Viral titer in supernatant was determined after 48 h of infection using plaque assay (*n* = 3). (**B**) Western blotting was done to confirm HuR silencing using anti-HuR antibody. Upper panel represents the densitometry values from three independent replicates, and the bottom panel depicts a representative image from one of the biological replicates. (**C**) Effect of HuR silencing on different SARS-CoV-2 VoCs. HEK-293T-ACE2 cells were transfected with either Nsp si or si HuR. Sixteen hours post-transfection, cells were infected with different SARS-CoV-2 VoCs at 0.1 MOI. Forty-eight hours post-infection, cell supernatant was collected to determine the viral titers by plaque assay (*n* = 3). (**D**) Representative images for plaque assay with VOCs. (**E**) Western blotting using anti-HuR antibody was performed for confirmation of HuR silencing. The upper panel is a representative of western from one of the biological sets of experiments, and the bottom panel represents densitometry values from three independent replicates. Two-way ANOVA, Nsp si vs si HuR = <0.001, VOCs = n.s. (0.721). (**F**) HuR KO was confirmed by western blotting using anti-HuR antibody. (**G**) Sequence alignment of HuR gene being targeted by the CRISPR gRNA. The blanks in KO sequence represent the deletion of the region between the two gRNAs. (**H**) WT HuR and HuR KO HEK-293T-ACE2 cells were infected with SARS-CoV-2 virus at 0.1 MOI and 48 h post-infection, positive-strand RNA levels were determined using primers specific for SARS-CoV-2 5′-UTR (*n* = 3). (**I**) WT HuR and HuR KO HEK-293T-ACE2 cells were infected with SARS-CoV-2 virus at 0.1 MOI and 48 h post-infection, viral titer in supernatant was determined using plaque assay (*n* = 3). (**J**) RNA levels of genomic 5′-UTR and those of subgenomic RNAs for spike and nucleocapsid were quantified using specific primers (*n* = 3). Student’s *t*-test was used for statistical analysis. Error bars represent SEM in all graphs. * = *P* < 0.05, ** = *P* < 0.01, *** = *P* < 0.001.

To study the role of HuR in the viral life cycle, ACE2-expressing HEK-293T cells were transfected with HuR short-interfering RNA (siRNA), which partially knocked down HuR expression by 70% (si HuR), or control siRNA (Nsp si), and infected with SARS-CoV-2 at a multiplicity of infection (MOI) of 0.1. The cells were harvested 48 h post-infection, and the viral titers in the supernatants of siHuR-treated cells and non-specific siRNA-treated cells were compared. We observed that siHuR treatment decreased the viral titer by more than 1 log (Fig. 1A and B), emphasizing its important role in the viral life cycle. Parallel experiments were performed to verify the effect of HuR on the life cycle of different viral variants. Silencing of HuR decreased the viral titers of all variants, but the effect was delayed with the alpha variant, with no difference observed after 24 h and a clear difference observed after 48 h (Fig. 1C through E). To confirm the role of HuR, we generated HuR knockout (KO) HEK-293T-ACE2 cells using a guide RNA targeting the HuR coding region. HuR KO was confirmed by western blotting using an anti-HuR antibody (Fig. 1F) and by sequencing (Fig. 1G). HuR KO increased cell proliferation slightly (see Fig. S2A at https://doi.org/10.6084/m9.figshare.31628611). We infected wild-type (WT) HuR-positive cells and HuR KO cells with WT SARS-CoV-2 at an MOI of 0.1 and examined differences in the supernatant viral titers and cellular SARS-CoV-2 RNA levels. Knocking out HuR reduced the cellular viral RNA levels by >80% (Fig. 1H) and decreased the viral titer by >2 logs (Fig. 1I). The abundances of SARS-CoV-2 subgenomic RNAs (sgRNAs) encoding the spike and nucleocapsid proteins decreased similarly to that of genomic RNA in the HuR KO cell line (Fig. 1J). The HuR KO cell line was also utilized to assess the impact of HuR complementation on viral RNA levels in SARS-CoV-2 variants of concern (VoCs). Overexpression of HuR was found to increase the viral RNA in all the VoCs (in varying amounts), suggesting an important role in viral life cycle (see Fig. S2B at https://doi.org/10.6084/m9.figshare.31628611). All further experiments relating to SARS-CoV-2 virus infection were performed using the WT virus (Isolate Hong Kong/VM20001061/2020).

### HuR bound the 5′-UTR of SARS-CoV-2

**Fig 2:**
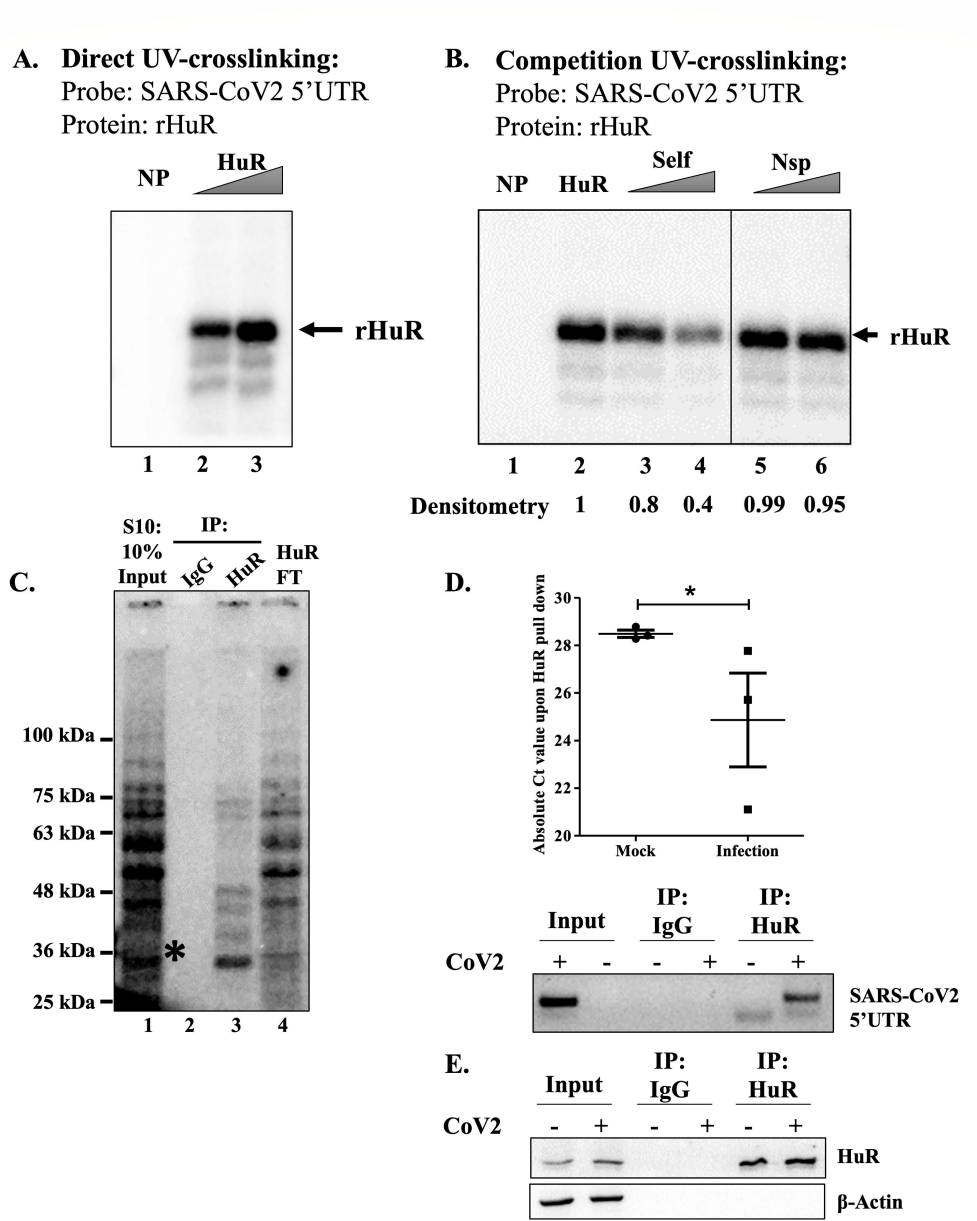
HuR binds to SARS-CoV-2 5′-UTR. (**A and B**) *In vitro* interaction of recombinant HuR with radiolabeled SARS-CoV-2 5′-UTR using UV-crosslinking assay. (**C**) UV crosslinking of S10 extract with radiolabeled SARS-CoV-2 5′-UTR, followed by immune-pulldown of HuR. The asterisk indicates the band at the predicted molecular weight of HuR. (**D**) *Ex vivo* interaction of HuR with CoV-2 5′-UTR in SARS-CoV-2 virus-infected cells was quantified upon immune-pulldown using anti-HuR antibody. IgG pulldown was used as a negative control to calculate the enrichment of SARS-CoV-2 5′-UTR in HuR pulldown. In the upper panel, absolute Ct values for SARS-CoV-2 5′-UTR from the HuR pulldown fraction were plotted (*n* = 3). The bottom panel denotes PCR amplification of CoV-2 5′-UTR (**E**) and the western blot for HuR pulldown in panel D. Student’s *t*-test was used for statistical analysis. * = *P* < 0.05, ** = *P* < 0.01, *** = *P* < 0.001.

To verify that HuR can bind the SARS-CoV-2 5′-UTR, we performed ultraviolet (UV)-crosslinking assays with a SARS-CoV-2 5′-UTR probe (transcribed *in vitro* with P^32^) and recombinant HuR protein. Direct UV crosslinking showed that HuR bound the labeled SARS-CoV-2 5′-UTR probe in a dose-dependent manner (Fig. 2A). The interaction was further confirmed by performing competitive UV-crosslinking experiments, where 100-fold and 150-fold excesses of unlabeled self-RNA showed increasing competition, which was not observed with an unlabeled non-specific RNA (Fig. 2B). These results confirmed the *in vitro* binding of HuR to the SARS-CoV-2 5′-UTR.

To verify the binding in cell lysates, we performed UV-crosslinking immunoprecipitation (IP) experiments wherein HuR was pulled down from the cytoplasmic S10 lysates of WT cells following UV crosslinking with the SARS-CoV-2 5′-UTR, using an anti-HuR antibody. Specific HuR binding to the SARS-CoV-2 5′-UTR was demonstrated by comparing the electrophoretic bands between experimental (after crosslinking and IP) and input samples. The band at approximately 36 kDa, which was absent from the flow-through beads, depicted the HuR band in the cell lysate (Fig. 2C). *Ex vivo* binding of HuR to the SARS-CoV-2 5′-UTR in infected cells was demonstrated by performing IP-reverse transcription (RT) experiments where HuR was pulled down using an anti-HuR antibody, and the associated SARS-CoV-2 5′-UTR was quantified using specific primers. The enrichment of SARS-CoV-2 5′-UTR in the HuR pulldown after 48 h of infection confirms the *ex vivo* interaction (Fig. 2D). IgG pulldown was used as a negative control for HuR pulldown. IL-6 RNA, which is a known interactor of HuR (18), was used as a positive control. We observed significant enrichment of IL-6 in the HuR pulldown fraction from mock cells (see Fig. S3A at https://doi.org/10.6084/m9.figshare.31628611). This interaction was reduced upon SARS-CoV-2 infection, which could be because of reduction in HuR levels (see Fig. S3B at https://doi.org/10.6084/m9.figshare.31628611) and sequestration of the remaining HuR by the viral RNA after 48 h of infection.

### HuR bound around the C241 site in the SARS-CoV-2 5′-UTR

To identify the HuR-binding site in the SARS-CoV-2 5′-UTR, we used catRAPID fragment software that considers the secondary structure when determining the binding affinity of a protein. HuR showed very high affinity for the region spanning nts 150–250, which corresponds to stem-loop 5 of the SARS-CoV-2 5′-UTR (Fig. S1C and D). Two HuR-binding motifs (199-GUUU-202 and 237-GUUU-240) were found within the region spanning nts 150–250, which overlapped with the loop regions of SL5A and SL5B, respectively. However, the catRAPID fragment showed lower affinity for the third 8-GUUU-11 motif, which base paired with the SL1 hairpin.

**Fig 3:**
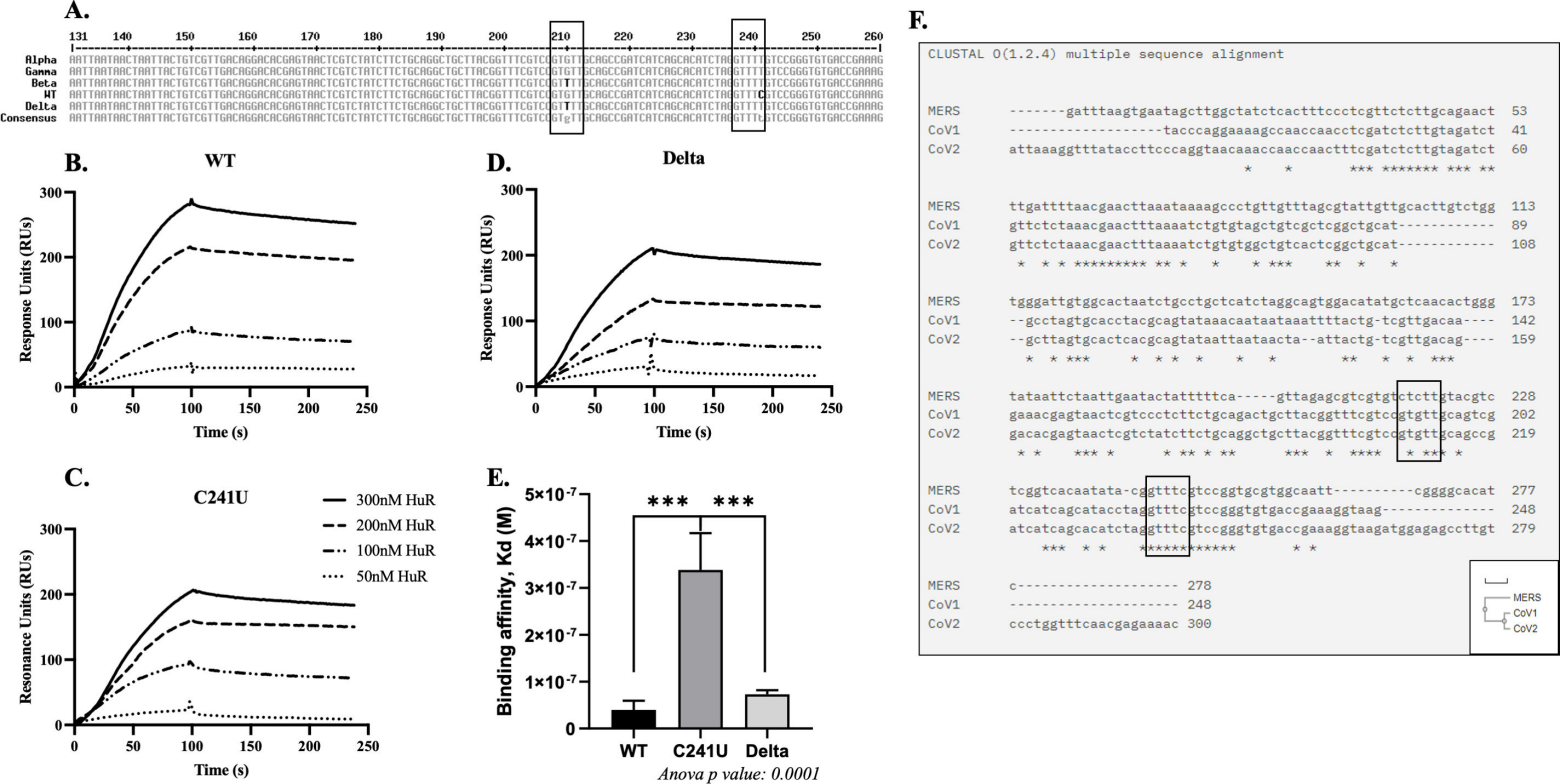
HuR binding site on SARS-CoV-2 5′-UTR. (**A**) Multiple sequence alignment of 5′-UTRs from WT and variant (alpha, beta, gamma, delta) SARS-CoV-2 genomes. (**B, C, D**) Sensorgrams for indicated biotinylated SARS-CoV-2 5′-UTR binding to recombinant HuR using SPR. Dotted lines represent different HuR concentrations used, as indicated in panel C. (**E**) Binding affinity of mutants obtained by SPR (*n* = 3). (**F**) Sequence alignment of representative 5′-UTRs of beta coronaviruses (MERS, SARS-CoV, and SARS-CoV-2). The highlighted boxes indicate the shortlisted HuR binding sites. Statistical analysis was performed using one-way ANOVA with *post hoc* Tukey’s test. * = *P* < 0.05, ** = *P* < 0.01, *** = *P* < 0.001.

To assess the impact of HuR binding, we evaluated the presence of mutations in the 5′-UTRs of different SARS-CoV-2 variants. We aligned the 5′-UTRs of the alpha, beta, gamma, and delta variants and found two mutations between them. The alpha variant acquired a C241U mutation, which was retained in the other successive variants. The beta and delta variants acquired another mutation, namely G210U (Fig. 3A).

We performed surface plasmon resonance (SPR) studies to analyze the effect of these mutations on the HuR-binding affinities (Fig. 3B through D). Incorporation of the C241U mutation decreased the binding affinity of HuR from 32.5 ± 13 nM (WT strain) to 338.6 ± 63 nM, whereas the incorporation of G210U along with C241U partially rescued the binding affinity to 72.9 ± 7.2 nM (Fig. 3E). These results could indicate that the second mutation (G210U) generated an additional binding site for HuR. These findings corroborate the variant data shown in Fig. 1, and the lower binding affinity of HuR to the alpha variant might explain its delayed effect. Furthermore, the 5′-UTRs of the alpha and gamma variants contained the same mutations, but the gamma variant did not show lesser HuR dependency at 24 h post-infection. The results could reflect other direct or indirect effects of HuR silencing. These data provide insights into the differential roles of HuR in SARS-CoV-2 variants due to the altered binding affinity of HuR. Furthermore, we analyzed the sequence conservation of these HuR binding sites at positions 210 and 241 among the beta coronaviruses, MERS, SARS-CoV, and SARS-CoV-2. The 5′-UTRs of SARS-CoV and SARS-CoV-2 exhibited high sequence conservation (Fig. 3F). The binding site around the 210 position showed a mutation from “C” in MERS to “G208 and G210” in SARS-CoV and SARS-CoV-2 (Fig. 3F). The presence of either C or G at these positions eliminates the presence of an HuR binding motif; however, the mutation G210U in the SARS-CoV-2 variants (Beta and Delta) generated an HuR binding site. Interestingly, we observed that the HuR binding site around the 241 position is completely conserved in the beta coronaviruses. The C241U mutation in SARS-CoV-2 variants further increased the binding affinity at this site. The evolutionary conservation of HuR binding site (237–240) in the 5′-UTR highlights its importance in the viral life cycle and propagation.

### HuR promoted translation from the SARS-CoV-2 genomic 5′-UTR

SARS-CoV-2 replication can be impacted through the regulation of many different steps in the viral life cycle. Because HuR bound to the SARS-CoV-2 5′-UTR (where translation of the genome begins), we investigated whether HuR regulates this step. To this end, we generated a luciferase reporter construct wherein the viral 5′-UTR was followed by the luciferase gene. Luciferase activities under different conditions revealed various degrees of SARS-CoV-2 5′-UTR translation.

**Fig 4:**
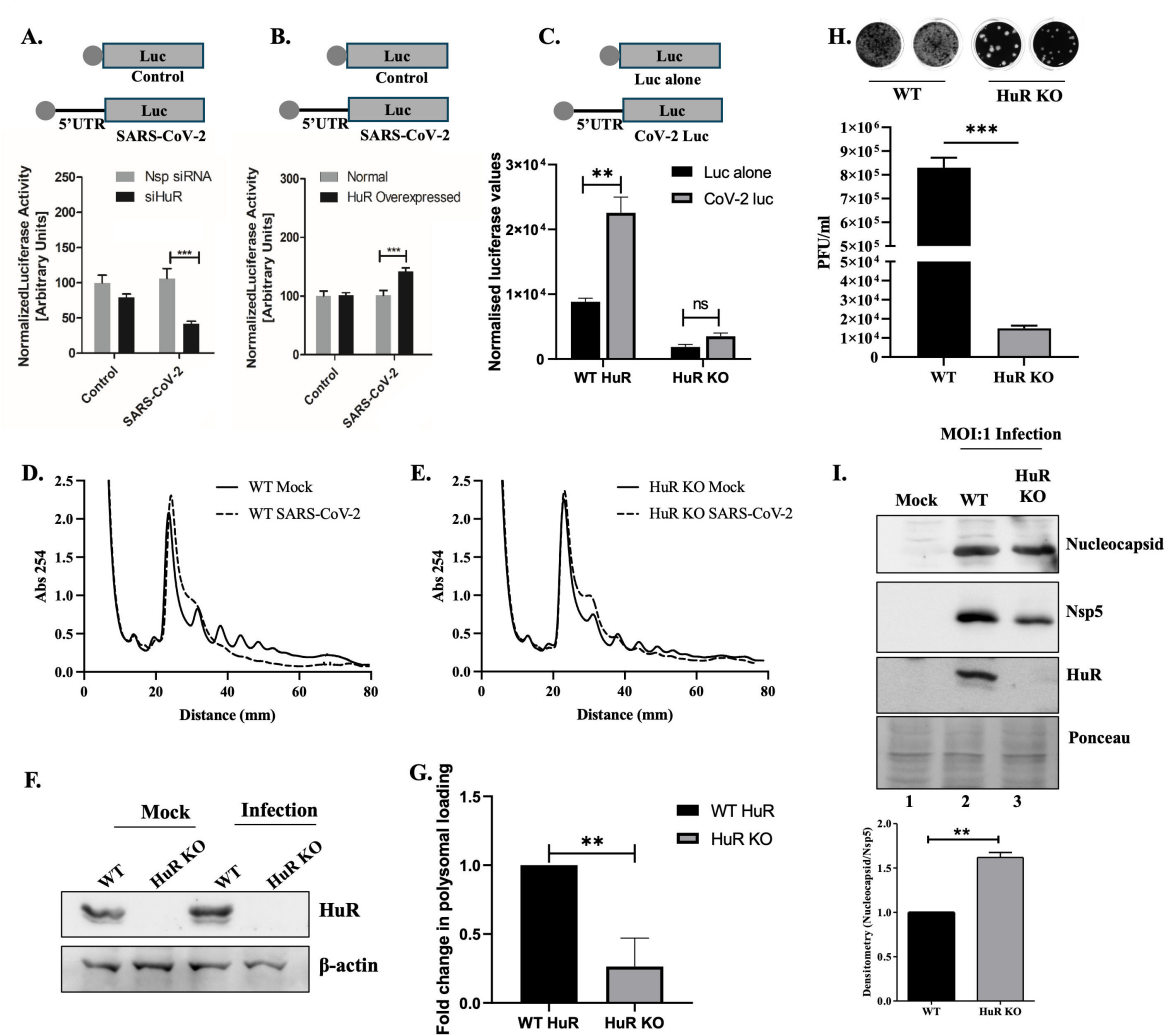
HuR promotes translation of non-structural protein by binding to SARS-CoV-2 genomic 5′-UTR. (**A**) A549 cells were transfected with either Nsp si or siHuR. Sixteen hours post-transfection, cells were transfected with either pGL3-SV40-Luc (Control) or pGL3-SV40-SARS-CoV-2 5′-UTR-Luc (SARS-CoV-2 Luc), depicted as schematics above the graphs, and harvested after 12 h of transfection and luciferase readings measured. pRLTK was co-transfected for transfection control and used for normalization of luciferase values. The effect of si HuR was calculated with respect to the luciferase in Nsp si condition (*n* = 3). (**B**) HuR overexpression was performed in place of silencing in the previous panel. The effect of overexpression was calculated with respect to luciferase in no-overexpression condition (*n* = 3). (**C**) WT HuR and HuR KO HEK-293T-ACE2 cells were transfected with either pGL3-SV40-Luc (Luc alone) or pGL3-SV40-SARS-CoV-2 5′-UTR-Luc (CoV-2 Luc), depicted as schematics above the graphs and luciferase values were measured 24 h post-transfection. The graph represents absolute values obtained upon normalization of luciferase readings with protein concentration (*n* = 3). (**D**) WT HEK-293T-ACE2 cells were infected with SARS-CoV-2 virus at 0.1 MOI, and 48 h post-infection, cells were harvested for sucrose gradient polysome fractionation. The lines represent the absorbance at 254 nm vs the distance from the top of gradient. (**E**) HuR KO HEK-293T-ACE2 cells were infected with SARS-CoV-2 virus at 0.1 MOI, and 48 h post-infection, cells were harvested for sucrose gradient polysome fractionation. The lines represent the absorbance at 254 nm vs the distance from the top of gradient. (**F**) Western blotting was performed for the lysates used for polysome fractionation using anti-HuR antibody. (**G**) RNA was isolated from monosome and polysomal fractions (separately) of infected lysates from panels D and E and the abundance of SARS-CoV-2 5′-UTR quantified by specific primers in real-time PCR. Polysomal loading was calculated as the ratio of RNA in polysome:monosomes (*n* = 3). (**H**) WT HuR and HuR KO HEK-293T-ACE2 cells were infected with SARS-CoV-2 virus at an MOI of 1.0 and 24 h post-infection, viral titer in supernatant was determined using plaque assay (*n* = 3). The top panel shows representative plaque assay images for the same. (**I**) Protein levels of nucleocapsid and Nsp5 were determined in the above experiment by western blotting using specific antibodies. Ponceau staining of the blot represents equal loading. The lower panel represents the average densitometry values after quantifying the intensities of nucleocapsid and Nsp5 bands (*n* = 3). Student’s *t*-test was used for statistical analysis. * = *P* < 0.05, ** = *P* < 0.01, *** = *P* < 0.001.

A549 cells were transfected with a 5′-UTR-luc construct in the context of either partial HuR knockdown (Fig. 4A) or HuR overexpression (Fig. 4B), and the luciferase activities were measured. To normalize the luciferase activities, the cells in each well were co-transfected with 2 ng of pRLTK (encoding Renilla luciferase), and dual-luciferase readings were taken after 8 or 12 h. Partial HuR knockdown reduced translation from the SARS-CoV-2 5′-UTR (Fig. 4A), and HuR overexpression increased translation (Fig. 4B). These results suggest that HuR promoted viral RNA translation by binding to the 5′-UTR. This effect was not limited to a lung cell line and was also observed in WT and HuR KO HEK-293T-Ace2 cells. The inclusion of the SARS-CoV-2 5′-UTR in the luciferase reporter construct showed increased luciferase activity in WT cells (lanes 1 and 2), suggesting that the presence of the SARS-CoV-2 5′-UTR might have recruited additional factors needed for enhancing cap-dependent viral translation. Furthermore, this increase was completely abrogated in HuR KO cells (lanes 3 and 4), implying the importance of HuR as the factor behind increased translation from the viral 5′-UTR. We quantified the luciferase RNA levels in this experiment and observed no significant difference, suggesting the impact of HuR on viral RNA translation (see Fig. S5A at https://doi.org/10.6084/m9.figshare.31628611). Notably, translation from the parental luciferase construct was reduced in HuR KO cells, which could represent an independent effect of HuR knockdown (Fig. 4C).

To confirm the role of HuR binding on translation, we generated additional luciferase constructs containing C241U mutation in the 5′-UTR. We transfected the WT and C241U mutant luciferase constructs in WT and HuR KO cells and observed that in WT cells, incorporation of C241U mutation reduced the translation from 5′-UTR to 20%. This reduction was partially rescued, with translation reaching 80% in HuR KO cells. This confirms that the 241 site is one of the major functional HuR binding sites which promotes translation from genomic 5′-UTR (see Fig. S4 at https://doi.org/10.6084/m9.figshare.31628611).

The role of HuR in SARS-CoV-2 translation was further studied by analyzing viral RNA loaded into the polysomes in WT and HuR KO cells. The reduction in polysomal peaks upon SARS-CoV-2 infection suggested that SARS-CoV-2 infection led to a global reduction in translation in WT cells (Fig. 4D). This reduction was compromised in HuR KO cells (Fig. 4E). We compared the global translation in WT and HuR KO cells by calculating the ratio of the area of polysomal to monosomal peak. Higher polysome:monosome ratio suggests higher translation. In all three experimental sets, we observed a slightly reduced polysome:monosome ratio in HuR KO cell line as compared to the WT, but the difference was not statistically significant (see Fig. S5B at https://doi.org/10.6084/m9.figshare.31628611). This suggests that there might be a slight reduction in global translation in HuR KO cells, which might also explain the lower luciferase value of the Luc-alone vector control in the HuR KO cells as compared to WT cells. We also calculated the polysome:monosome ratio in infected WT and HuR KO cells, where we found a higher ratio in the HuR KO cells (see Fig. S5C at https://doi.org/10.6084/m9.figshare.31628611). This further confirms that the global translational reduction upon SARS-CoV-2 infection is much higher in WT cells than in HuR KO cells. Of note, we cannot compare the ratio of area under the curve in mock and infected cells because of the major differences in the polysome profiles in these conditions such as the transition phase from monosome to polysomes (Fig. 4D and E). Furthermore, to assess the polysomal loading of the viral RNA, RNA was isolated from monosome and polysome fractions separately, and the abundances of the SARS-CoV-2 5-UTR were quantified by real-time polymerase chain reaction (PCR) analysis using specific primers. The RNA loading to polysomes was calculated as a ratio of RNA in polysome:monosomes. We found increased polysomal loading of SARS-CoV-2 RNA as compared to the actin RNA in WT infected cells, suggesting the preferential loading of viral RNAs in the polysome even after global translational reduction (see Fig. S5D at https://doi.org/10.6084/m9.figshare.31628611). HuR knockout led to a 60%–70% decrease in SARS-CoV-2 RNA loading into polysomes as calculated by ratio of RNA loaded in polysome: monosomes (Fig. 4F and G). This finding confirms the important role of HuR in promoting translation from SARS-CoV-2 5′-UTR. We observed that HuR KO led to decreased viral genomic and sgRNA production (Fig. 1J). Since they are independent RNA species with only the first 69 nts overlapping in the 5′-UTR (19–21), we examined the effect of HuR KO on translation on the sg 5′-UTR and genomic 5′-UTR. To this end, we infected WT and HuR KO cells with SARS-CoV-2 at an MOI of 1.0 and harvested the cells and culture supernatants after 24 h. We observed a >10-fold reduction in viral titers in HuR KO cells (Fig. 4H). We quantified the expression levels of non-structural and structural proteins (produced by the translation of genomic and subgenomic 5′-UTRs, respectively) in WT and HuR KO cells by western blot analysis. The Nsp5 protein represents genomic translation, and the nucleocapsid protein represents translation from the subgenomic RNA. The Nsp5 levels reduced upon HuR KO, corroborating the effect on genomic translation (Fig. 4I). We also found a similar effect of HuR KO on another non-structural protein, Nsp1 (see Fig. S5E at https://doi.org/10.6084/m9.figshare.31628611), confirming the role of HuR in mediating polyprotein translation from the 5′-UTR and ruling out the effect on stability of independent non-structural proteins. Interestingly, the expression levels of nucleocapsid protein did not change even when the subgenomic RNA levels reduced by the same extent as the genomic RNA upon HuR KO, pointing toward higher translation per RNA molecule (Fig. 1J). Similar results were observed for another structural protein, spike (see Fig. S5F at https://doi.org/10.6084/m9.figshare.31628611). These findings suggest that HuR binding to genomic 5′-UTR promotes the production of non-structural proteins. This effect seems to be reversed upon binding to the subgenomic RNA, possibly because of reduced HuR-binding sites in the subgenomic 5′-UTR.

To study subgenomic RNA translation, we performed capped *in vitro* transcription of subgenomic SARS-CoV-2 5′-UTR (nts 1–70) followed by luciferase RNA and used *in vitro*-transcribed luciferase RNA alone as a control. These RNAs were transfected into WT and HuR KO HEK-293T-ACE2 cells, and luciferase activities were measured at 8 h post-transfection. As observed previously, transfecting the parental luciferase reporter RNA alone exhibited lower readings in the HuR KO cell line. This decrease was rescued for sg 5′-UTR-luc, suggesting the increase in translation from the sg 5′-UTR as compared to the luc alone in the HuR KO cell line (see Fig. S5F at https://doi.org/10.6084/m9.figshare.31628611). These data explain the increased ratio of structural to non-structural proteins in the HuR KO cell line and provide evidence of differential translational regulation of SARS-CoV-2 genomic RNA and sgRNA by a host protein.

### HuR regulated SARS-CoV-2 translation by assisting binding of the polypyrimidine tract-binding (PTB) protein

**Fig 5:**
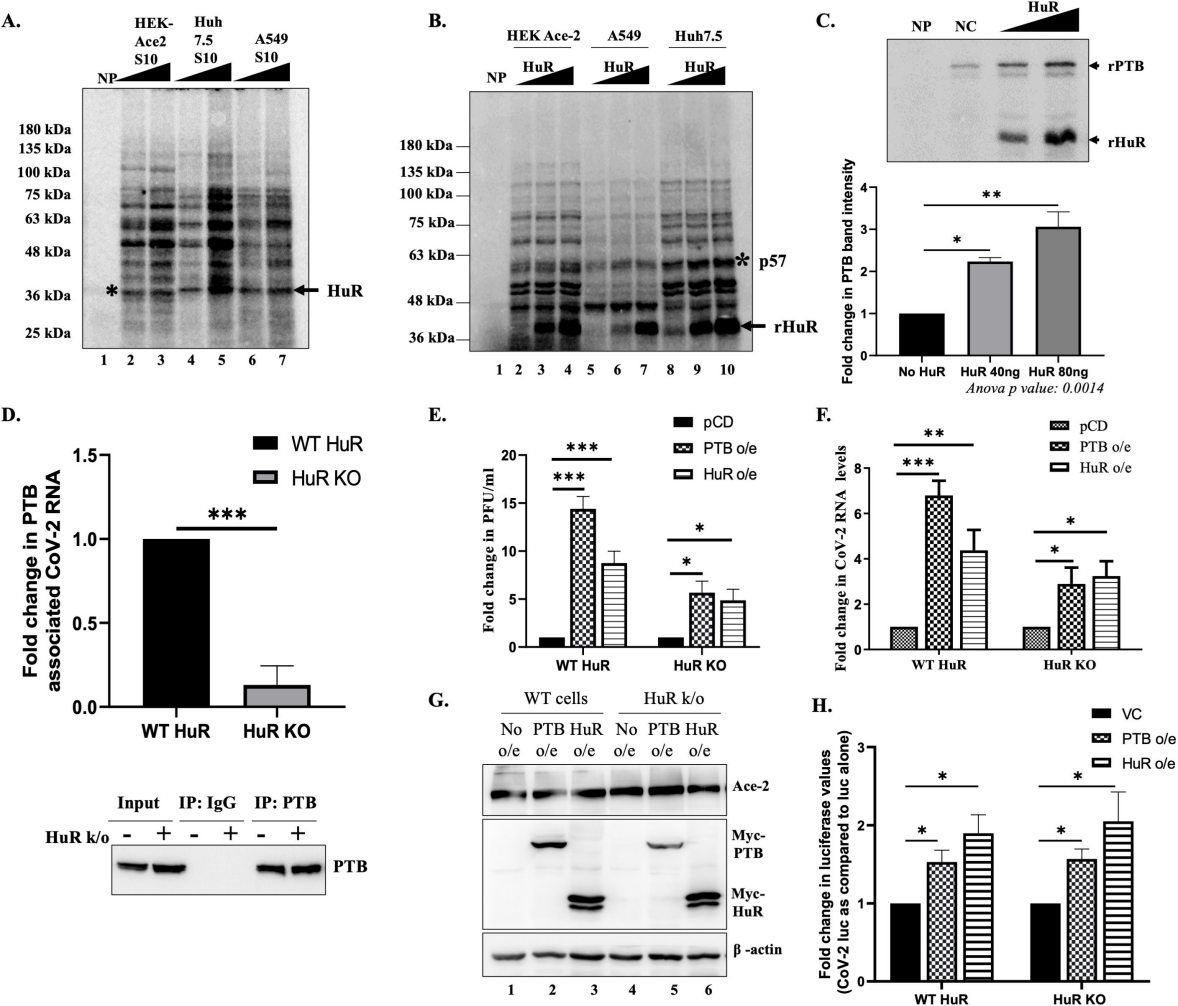
HuR assists PTB binding to 5′-UTR to promote translation. (**A**) UV-crosslinking profiles with increasing amounts of S10 extracts of different cell lines as indicated. Asterisk indicates the band at the size of HuR. (**B**) UV crosslinking was performed with S10 extracts of different cell lines and increasing amounts of HuR. Asterisk indicates the band at the size of PTB. (**C**) Competition UV crosslinking was performed with a constant amount of PTB and increasing amounts of recombinant HuR. The lower panel represents the densitometry of PTB band intensity (*n* = 3). Statistical analysis was performed using one-way ANOVA with *post hoc* Tukey’s test. (**D**) HEK-293T-ACE2 with WT and HuR KO were infected with 0.1 MOI of SARS-CoV-2. Forty-eight hours post-infection, cells were harvested and PTB was immunoprecipitated. The associated RNA was quantified by real-time PCR using SARS-CoV-2 5′-UTR specific primers (upper panel). IgG pulldown was used as a negative control to calculate the enrichment of SARS-CoV-2 5′-UTR in PTB pulldown. Fold change of enrichment was plotted as compared to the enrichment in WT cells (*n* = 3). The immune-pulldown was confirmed by western blot using anti-PTB antibody (lower panel). Student’s *t*-test was used for statistical analysis. (**E**) WT and HuR KO HEK-293T-ACE2 cells were transfected with myc-tagged HuR and PTB overexpression constructs. Sixteen hours post-transfection, cells were infected with SARS-CoV-2 at 0.1 MOI. Cell supernatant was collected after 48 h of infection, and viral titers were determined using plaque assay (*n* = 3). Statistical analysis was performed using two-way ANOVA with *post hoc* Tukey’s test. (**F**) Viral RNA levels were quantified in cells from the previous experiment (*n* = 3). Statistical analysis was performed using two-way ANOVA with *post hoc* Tukey’s test. (**G**) Western blotting was performed using anti-myc antibody confirmation of overexpression. (**H**) WT and HuR KO HEK-293T-ACE2 cells were transfected with myc-tagged HuR and PTB overexpression constructs. Sixteen hours post-transfection, cells were transfected with pGL3-SV40-Luc (Luc alone) or pGL3-SV40-SARS-CoV-2 5′-UTR-Luc (CoV-2 Luc) and harvested after 24 h of transfection and luciferase readings measured (*n* = 3). Student’s *t*-test was used for statistical analysis. * = *P* < 0.05, ** = *P* < 0.01, *** = *P* < 0.001.

To further investigate the mechanism of action of HuR, we analyzed the binding of different cellular proteins to the 5′-UTR of SARS-CoV-2. We purified cellular S10 extracts from three different cell lines to rule out a cell line-specific effect (HEK-293T-ACE2, A549, and Huh7.5) and analyzed their binding to the SARS-CoV-2 5′-UTR by performing UV-crosslinking experiments. We observed that multiple proteins bound to the 5′-UTR in a dose-dependent manner (Fig. 5A). The band marked with an asterisk had a mass that corresponded to the size of HuR, as verified in UV-IP experiments (Fig. 2C).

We checked whether the addition of exogenous HuR to the S10 lysate would alter the binding to any other protein and observed a band, approximately 57 kDa in size, whose intensity increased with increasing concentrations of HuR (Fig. 5B). We previously characterized PTB as an important protein with a band in this size range that is involved in an interplay with HuR for binding to viral UTRs (9). Therefore, we purified recombinant PTB and HuR proteins and performed competitive-crosslinking experiments with increasing HuR concentrations, while keeping the PTB concentration constant. While PTB bound the SARS-CoV-2 5′-UTR even in the absence of HuR, HuR binding promoted PTB binding to the SARS-CoV-2 5′-UTR in a concentration-dependent manner, corroborating our previous data (Fig. 5C). We analyzed whether this cooperation occurs *ex vivo* in cells by performing IP-RT experiments, where PTB was immunoprecipitated from cells expressing WT HuR and HuR KO cells (Fig. 5D). Thereafter, the associated 5′-UTR was quantified by real-time PCR. The reduction in PTB-associated viral RNA in the absence of HuR (Fig. 5D, upper panel) confirms that HuR functions by promoting the binding of PTB.

Because HuR and PTB are primarily nuclear proteins that relocalize to the cytoplasm upon infection with different viruses, we checked their localization at different time points following infection by conducting immunofluorescence and nuclear-cytoplasmic fractionation experiments (Fig. S6 B through E). By nuclear-cytoplasmic fractionation assay for PTB, we observed increased cytoplasmic localization after 48 h of infection. In the immunofluorescence assays, HuR localized to the nucleus in mock-infected cells, and we did not observe HuR cytoplasmic relocalization when HEK-293T-ACE2 cells were infected with SARS-CoV-2 at different time points (see Fig. S6C at https://doi.org/10.6084/m9.figshare.31628611). Nuclear-cytoplasmic fractionation experiments revealed that a fraction of HuR localized to the cytoplasm and that its cytoplasmic abundance did not increase upon SARS-CoV-2 infection (see Fig. S6D and E at https://doi.org/10.6084/m9.figshare.31628611). The cytoplasmic HuR is sufficient for promoting PTB binding and influencing viral translation. Interestingly, we observed a decrease in cytoplasmic HuR after 48 h of infection, which justifies the overall HuR reduction observed in Fig. S3B at https://doi.org/10.6084/m9.figshare.31628611. This reduction might be a part of the cellular response to viral infection.

We examined the role of PTB in the viral life cycle and observed a significant decrease in viral RNA levels upon PTB knockdown (see Fig. S6A at https://doi.org/10.6084/m9.figshare.31628611). We then examined whether PTB overexpression could rescue the decrease in viral titer in HuR KO cells. PTB overexpression increased the viral titer by more than 1 log in both the WT HuR and HuR KO cell lines (Fig. 5E). PTB overexpression demonstrated higher increase in viral RNA levels and viral titers in WT HuR cell line as compared to the HuR KO cell line, suggesting that the effect of PTB on viral life cycle might be mediated through HuR (Fig. 5F and G). The increase in viral RNA and titers upon PTB overexpression was similar to HuR overexpression. Luciferase constructs were used to examine whether these effects were mediated through translational regulation. PTB overexpression increased luciferase activity in WT and HuR KO cell lines (Fig. 5H). Slightly higher (1.5- vs 2-fold) increase in translation was observed upon HuR overexpression. These results suggest that the presence and binding of HuR to the SARS-CoV-2 5′-UTR could guide PTB binding and thereby promote viral translation. However, both HuR and PTB can bind directly to the 5′-UTR, and the same effect on translation was observed by increasing PTB expression in cells, which would increase its binding propensity and affinity because of the increased abundance. These findings suggest a model where during initial hours of infection, PTB and HuR are both present in low amounts in the cytoplasm. PTB has the ability to directly bind the SARS-CoV-2 5′-UTR, but the presence of HuR enhances this binding affinity to promote translation. Thereafter, by some cellular response, cytoplasmic HuR levels are reduced, and PTB is relocalized to cytoplasm at this time to make up for the reduction in HuR levels and continue the viral life cycle.

### HuR can potentially be targeted for antiviral development

Because HuR played an important role in guiding SARS-CoV-2 viral translation by binding to viral RNA, we explored whether targeting HuR could be a potential antiviral strategy.

**Fig 6:**
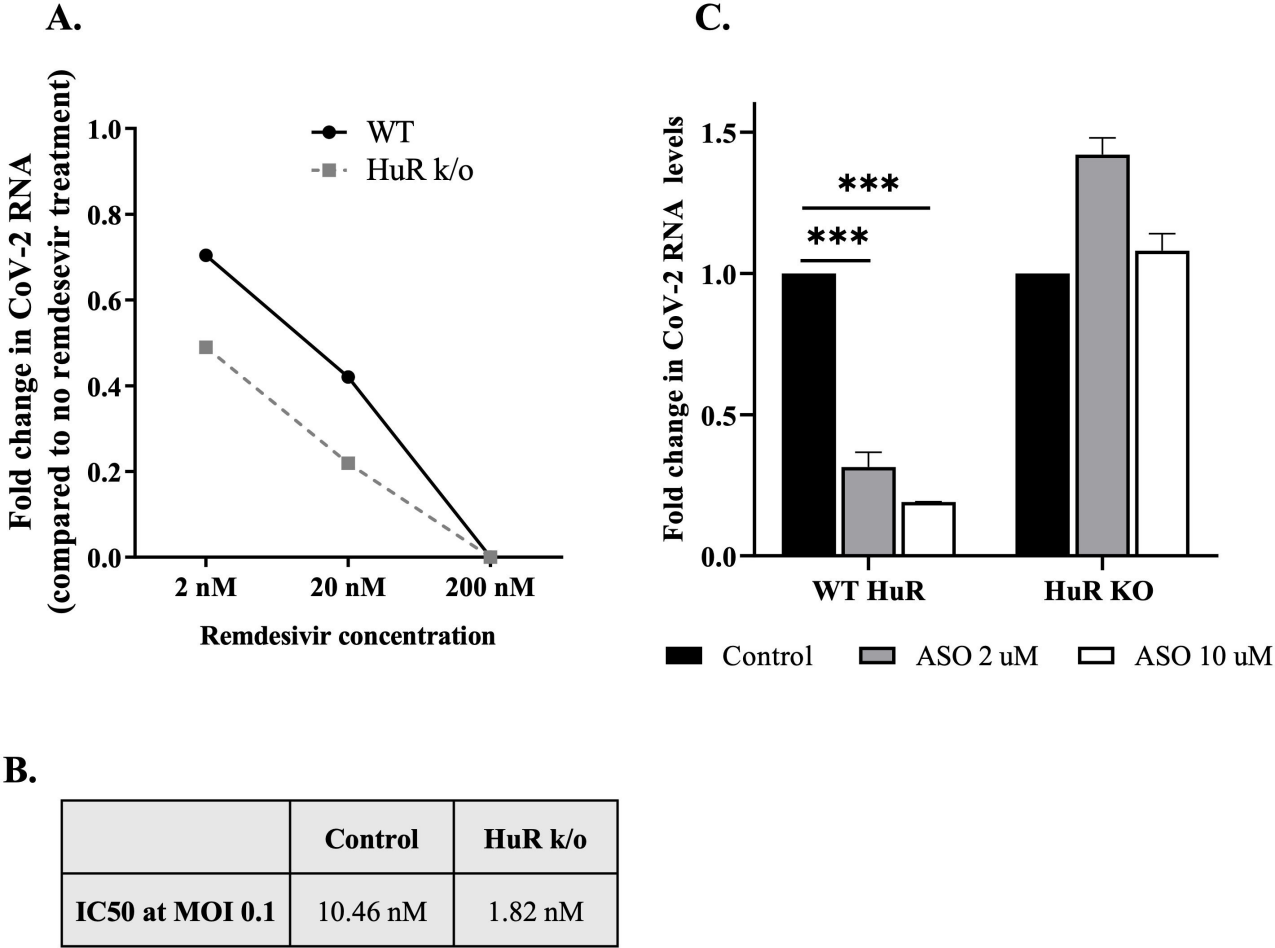
Therapeutic significance of the role of HuR in the SARS-CoV-2 life cycle. (**A**) Effect of HuR KO on remdesivir sensitivity of viral infection. WT and HuR KO HEK-293T-ACE2 cells were infected with SARS-CoV-2 virus at an MOI of 0.1, and the mentioned concentrations of remdesivir were added after 1 h of virus inoculation. Cells were harvested after 48 h of treatment and RNA isolated to detect SARS-CoV-2 5′-UTR. (**B**) Table for IC_50_ of remdesivir for CoV-2 infection in WT and HuR KO cell lines. (**C**) Effect of ASOs targeting HuR binding site on viral titers. WT and HuR KO HEK-293T-ACE2 cells were transfected with the mentioned concentrations of ASO, and 12 h post-transfection, cells were infected with SARS-CoV-2 virus at 0.1 MOI. Forty-eight hours post-infection, cells were harvested, and SARS-CoV-2 RNA was quantified using real-time PCR (*n* = 3). Statistical analysis was performed using one-way ANOVA with *post hoc* Tukey’s test. * = *P* < 0.05, ** = *P* < 0.01, *** = *P* < 0.001.

We investigated if reducing total HuR in the cells could alter the activity of remdesivir in viral inhibition. We used a concentration gradient of remdesivir and examined the decrease in viral RNA in WT and HuR KO cells. We found that 200 nM remdesivir completely inhibited viral RNA production in both cell lines; however, at lower remdesivir concentrations, the HuR KO cell line showed substantially lower viral RNA levels (Fig. 6A). The half-maximal inhibitory concentration (IC_50_) of remdesivir was calculated based on the resulting data, and we found that the IC_50_ was 10.46 nM in WT cells but only 1.82 nM in HuR KO cells (Fig. 6B). These data suggest the potential of targeting HuR in addition to remdesivir for reducing viral levels in the cells.

We further tried a strategy to reduce HuR binding to only SARS-CoV-2 5′-UTR. Recently, ASOs directed against different regions of SARS-CoV-2 RNA have been demonstrated to inhibit SARS-CoV-2 replication in cell culture and in animal models (22). Of the ASOs used, ASO5 targeted the HuR-binding site (encompassing the 241 site) and reduced SARS-CoV-2 RNA levels. Therefore, we investigated the activity of ASO5 using WT and HuR KO cell lines. As observed previously, ASO5 transfection reduced viral RNA levels in a dose-dependent manner in WT cells. However, such a decrease was not observed in the HuR KO cell line (Fig. 6C), strengthening the evidence supporting the location of the HuR-binding site, its role in the viral life cycle, and strategizing it as a potential antiviral target.

## DISCUSSION

Host proteins are important factors that regulate the viral life cycle. RNA-binding proteins bind to viral RNAs at different stages of their life cycles, thereby modulating their cellular interactions. RBP screening has identified common proteins involved in the life cycle of multiple viruses. HuR is one such protein that has been shown to regulate the translation and replication of viruses, including hepatitis C virus (9), coxsackievirus (11), and Sindbis virus (13). We studied the role of this important protein in regulating the SARS-CoV-2 life cycle. We found that HuR bound to the viral 5′-UTR and regulated its life cycle by promoting genomic 5′-UTR-mediated translation. It did so by promoting the binding of another RBP, PTB, which can enhance ribosome recruitment to the viral 5′-UTR. HuR binding occurred near the 241 site in the 5′-UTR, which is mutated in some SARS-CoV-2 variants of concern. This mutation alters the binding affinity for HuR and the reduces the dependency of the alpha variant on HuR, which could be one of the mechanisms behind the longer generation time and hence lesser transmission of the alpha variant as compared to the delta variant (23). Knocking out HuR increased the sensitivity of infected cells toward remdesivir-mediated viral inhibition. This suggests the potential of HuR inhibition as a new antiviral strategy. The HuR-binding site lies in the target region of antisense oligo ASO5 (22), which inhibits the viral life cycle and therefore would be an attractive candidate for such strategies. We further addressed the HuR-mediated translational regulation of SARS-CoV-2 genomic and sgRNAs. We showed that while HuR promoted translation from the genomic 5′-UTR, its knockout promoted sg 5′-UTR translation. Previous reports have demonstrated the translational regulation by viral proteins such as Nsp1 (24, 25) and ORF6 (26), and we propose that HuR could represent the cellular arm of such regulation. The genomic and subgenomic 5′-UTRs vary in length. The subgenomic UTR harbors one of the four predicted HuR binding sites (8–10), and that site resides in the stem region of the secondary structure, while the other three sites, which are present in the full-length UTR, reside in the loop region in the secondary structure (16). This could be responsible for the differential effect of HuR binding on genomic and subgenomic 5′-UTR. Furthermore, it could also affect the PTB recruitment capability of HuR, altering the translation regulation at the initial stage of viral infection. Both genomic and subgenomic RNA have been found to bind to HuR (ELAVL1) and PTB (PTBP1) in genome-wide interaction screens (27). In fact, at the late time point of SARS-CoV-2 infection cycle, PTB binding to subgenomic RNA was found to be higher than the genomic RNA. Combining this scheme with our translational results suggests that the cell could remodel PTB to act as an anti-viral factor at the later stages of viral infection.

HuR does not relocalize to the cytoplasm upon SARS-CoV-2 infection in HEK-293T-ACE2 cells, but the small amount of HuR present in the cytoplasm due to nuclear-cytoplasmic shuttling might be sufficient to promote PTB binding, which appears to relocalize to the cytoplasm upon infection. This possibility was supported by our competition-crosslinking assays, where a small quantity of HuR greatly enhanced PTB binding. We do not rule out the independent effects of HuR and PTB binding to the SARS-CoV-2 5′-UTR on viral translation or replication, nor the involvement of other cellular factors that regulate HuR and PTB during viral infection; these possibilities can be explored in future studies. Furthermore, HuR is known to alter the stability of cellular transcripts. The cytokine storm and disease severity observed upon SARS-CoV-2 infection could be regulated by HuR by virtue of its binding to mRNAs encoding pro-inflammatory cytokines such as interleukin (IL)-6 and IL-8 (28, 29). We also observed that HuR levels decreased following SARS-CoV-2 infection. The regulatory mechanism could be interesting, because miRNAs, especially miR-125b-5p, have been shown to target HuR translation (30). Several small molecules and drugs have been designed which abrogate HuR function by inhibiting the binding of HuR to its cellular transcripts (31, 32). Many of them have shown excellent efficacy in animal models, and some are in human trials as well. Suramin is one such FDA-approved drug that is prescribed for the treatment of African sleeping sickness and is effective against viruses like HIV, Chikungunya, and Ebola (33). It has also been shown to be efficacious in preventing SARS-CoV-2 infection by pathways including blockage of viral entry, inactivation of nucleocapsid protein, and inhibition of the polymerase (34–37). Inhibition of HuR and its role in viral translation could be another potential mechanistic arc in its antiviral activity. Such drugs are important targets to be explored for either their individual or synergistic repurposing as antivirals for SARS-CoV-2 infection.

Taken together, our observations provide important insights and open multiple avenues for targeting the SARS-CoV-2 life cycle by modulating an important host factor, HuR. Targeting HuR through ASOs could be an important anti-viral strategy along with remdesivir. It would prevent HuR binding to the genomic 5′-UTR, leading to decreased structural protein translation and thereby help in clearing SARS-CoV-2 infection.

## MATERIALS AND METHODS

### Plasmids and constructs

To make the template for *in vitro* transcription, the SARS-CoV-2 5′-UTR was amplified with 5′-AATTGCTAGCATTAAAGGTTTATACCTTCCCAGGTA-3′ and 5-′AATTAAGCTTCTCGTTGA AACCAGGGACAAG-3′ from pCBB 5′-UTR (kind gift from Dr. Milan Surjit, THSTI) and subcloned downstream of the T7 promoter in the NheI and HindIII cloning sites of pcDNA3.1+. For the luciferase assay, SARS-CoV-2 5′-UTR was amplified with 5′-AATTAAGCTTATTAAAGGTTTATACCTTCCCAGGTAAC-3′ and 5′-AATTCCATGGTTCTCGTTGAAACCAGGGACAAG-3′ to subclone into the pGL3 control vector. pcDNA3.1-myc-HuR and pET28a-HuR were generated as described previously. pcDNA3.1-myc-PTB was a kind gift from Dr. Douglas Black’s laboratory.

### Cell lines and transfection

HEK-293T cells expressing ACE2 (obtained from BEI Resources), A549 (ATCC) lung epithelial cells, and Vero E6 cells were grown in DMEM media (Gibco) supplemented with 10% FBS (Gibco). For luciferase assays in A549 cells, the day before transfection, 1.3 × 10^5^ cells were distributed per well of a 24-well tissue culture plate. Before transfection, DMEM was replaced with Opti-MEM I reduced serum media (Gibco). Lipofectamine 2000 (Invitrogen) was used for all transfection experiments as per manufacturer’s protocol. To check the effect of HuR overexpression, we co-transfected 250 ng of pGL3-SARS-CoV-2 5′-UTR with 250 ng of pcDNA3.1 or pCDNA3.1-HuR. pGL3 control vector was used for the control set. Two nanograms of pRLTK was also co-transfected in each well to normalize the firefly luciferase activity. For the HuR knockdown experiment, we transfected 150 nM of either DsiHuR: 5′AUUUCUGAAUCUGUGACGCAAGAAT 3′ (IDT) or nonspecific (Eurogentec) siRNA 24 h prior to luciferase construct transfection.

### Virus stock preparation and infection

Virus stocks were procured from BEI Resources, NIAID, NIH and maintained by the Viral BSL-3 repository at IISc. WT virus: Isolate Hong Kong/VM20001061/2020, NR-52282; Alpha variant (Lineage B.1.1.7): Isolate hCoV19/England/204,820,464/2020, NR-54000; Beta variant (Lineage B.1.351): Isolate hCoV-19/USA/MD-HP01542/2021, NR-55282; Gamma variant (Lineage P.1): Isolate hCoV-19/Japan/TY7-503/2021, NR-54982; Delta variant (Lineage B.1.617.2): Isolate hCoV-19/USA/PHC658/2021, NR-55611. Vero E6 cells were used to propagate and titrate all the viruses as described before (38). For virus experiments, HEK-293T-ACE2 cells were seeded in 24-well plates. The cells were transfected with 150 nM of Nsp si or si HuR using Lipofectamine 2000 as per the manufacturer’s protocol. Sixteen hours after transfection, cells were infected with virus stock at an MOI of 0.1 or 1 as described.

### Dual luciferase assay

Luciferase assay was done using the dual luciferase assay kit (Promega) according to manufacturer’s protocol. For HuR overexpression and siHuR experiments, assays were done 12 and 8 h post-transfection of luciferase constructs, respectively. Luciferase reading was taken with a Glomax Luminometer (Promega).

### Protein purification

*Escherichia coli* BL21 cells were transformed with pET28a containing His-tagged HuR. Recombinant protein was purified with Ni-NTA agarose. The details of purification was described earlier. Briefly, the expression of HuR was induced at an optical density of 0.6 at 600 nm with 0.5 mM isopropyl-1-thio-β-D-galactopyranoside for 4 h. The cells were pelleted and resuspended in lysis buffer (50 mM Tris, pH 7.5, 300 mM NaCl, 0.1 mM phenylmethylsulfonyl fluoride) containing 1× bacterial protease inhibitor (Sigma) and lysed by sonication on ice. The supernatant obtained thereafter was incubated with Ni-NTA slurry for 3 h, and bound protein was eluted with 500 mM imidazole after washing the beads with lysis buffer containing 40 mM imidazole. The eluted protein was dialyzed in 10 times the volume of dialysis buffer (25 mM Tris, pH 7.4, 100 mM KCl, 7 mM β-mercaptoethanol, 10% glycerol), aliquoted, and stored in −70°C.

### *In vitro* transcription

The pcDNA3.1-SARS-CoV-2 5′-UTR was linearized with HindIII digestion and purified with the phenol-chloroform method. The linearized DNA was used as a template for synthesis of RNA using T7 RNA polymerase (ThermoScientific) and ^32^P uridine triphosphate. The transcription reactions were carried out under standard conditions at 37°C for 1.5 h. After alcohol precipitation, RNA was resuspended in nuclease-free water. For radiolabeled RNA, 1 μL of the prepared RNA was spotted onto DE81 filter paper, washed with phosphate buffer, dried, and incorporated radioactivity was measured using a scintillation counter.

### UV-induced crosslinking of RNA and protein

UV-induced cross-linking was carried out as described previously. Briefly, ^32^P-labeled RNA probes were allowed to form complexes with recombinant HuR in RNA binding buffer (5 mM HEPES, pH 7.6, 25 mM KCl, 2 mM MgCl_2_, 3.8% glycerol, 2 mM dithiothreitol [DTT], and 0.1 mM EDTA) and then UV irradiated at 254 nm for 20 min. The mixture was treated with 50 µg of RNase A (Sigma), separated on an SDS-10% polyacrylamide gel (SDS-PAGE), and band analyzed by phosphorimaging. The 1–300 nts of SARS-CoV-2 genome were used as the SARS-CoV-2 5′-UTR to protect the secondary structures in the UTR. The multiple cloning site of the pGEX vector was transcribed and used as non-specific RNA.

### Surface plasmon resonance

Surface plasmon resonance spectroscopy was performed using a BIAcore3000 optical biosensor (GE Healthcare Lifescience) to study the binding kinetics of HuR with SARS-CoV-2 5′-UTR RNA. Biotin-labeled SARS-CoV-2 5′-UTR RNA was immobilized on streptavidin-coated sensor chips (GE Healthcare Lifescience) to a final concentration of 300 resonance units (RU)/flow cell. RNA-protein interactions were carried out in a continuous flow of Tris buffer (25 mM Tris [pH 7.5], 100 mM KCl, 7 mM β-mercaptoethanol, and 10% glycerol) at 25°C at a flow rate of 10 µL/min. Increasing concentrations of HuR protein were loaded on the biosensor chip for 100 s (characterized as the association phase), followed by a dissociation phase of 300 s with buffer alone. For normalizing background non-specific interaction, a blank surface without any RNA was used for simultaneous injections of the sample during the experiment. BIAevaluation software (version 3.0) was used to determine the on rate, *k*on (M^−1^ s^−1^), and off rate, *k*off (s^−1^), using a 1:1 Langmuir binding model. The binding affinity, *Kd,* was determined using the following equation: *Kd* = *k*off/*k*on.

### Plaque-forming assay

Plaque assay was performed in Vero E6 cells in 12-well plates. Initially, 0.5 × 10^6^ cells were seeded to give 90% confluency. After 12–16 h, cells were incubated with 100 µL of increasing dilution of supernatant obtained from Nsp si or si HuR treated ACE2-HEK-293T cells infected with SARS-CoV-2 or supernatant from mock-infected cells. After 1 h of incubation with the cells at 37°C, viral inoculum was removed and cells overlaid with DMEM containing 0.8% agarose and kept at 37°C. After 48 h of incubation, cells were fixed with 4% paraformaldehyde for 1 h and stained with crystal violet for 20 min. The overlay was removed and the number of plaques counted. The following calculation was used to determine PFU/mL: PFU/mL = no. of plaques ÷ (dilution factor × vol of virus inoculum in mL).

The supernatant from one experiment was used in a technical duplicate to examine the number of plaques. This was repeated for three biological replicates, and the average and standard deviation were calculated to plot the figures.

### Immunofluorescence

For immunofluorescence staining, HEK-293T-ACE2 cells were seeded in a 24-well plate on coverslips coated with poly-L-lysine for 14–16 h, followed by infection with SARS-CoV-2 virus at an MOI of 0.1. After the desired time of infection, cells were washed twice with 1× PBS and fixed using 4% formaldehyde at room temperature for 20 min. After permeabilization by 0.1% Triton X-100 for 5 min at room temperature, cells were incubated with 3% BSA at 37°C for 1 h, followed by incubation with the indicated antibody for 2 h at 4 °C and then detected by Alexa-633-conjugated anti-mouse or Alexa-488-conjugated anti-rabbit secondary antibody for 30 min (Invitrogen). Images were taken using a Zeiss microscope, and image analysis was done using the Zeiss LSM or ZEN software tools.

### HuR knockout cell line preparation

The guide RNA targeting HuR sequence was designed using Zhang lab software. These gRNAs were cloned in the pSpCas9(BB)−2A-GFP (PX458) vector as per the described protocol. The clones were transfected into HEK-293T-ACE2 cells, and 48 h post-transfection, the cells expressing GFP were sorted as one cell per well in a 96-well plate. These cells were grown and analyzed for HuR knockout using western blotting and confirmed through sequencing. For control, untransfected cells were also sorted as one cell per well in parallel, and one of the clones expressing ACE2 receptor was used. Sequences are as follows: for gRNA1, ACCACATGGCCGAAGACTGC; for gRNA2, CTTATTCGGGATAAAGTAGC.

### PTB knockdown cell line preparation

The guide RNA targeting the PTB sequence was designed using Zhang lab software. These gRNAs were cloned in the pSpCas9(BB)-2A-GFP (PX458) vector as per the described protocol. The clones were transfected into HEK-293T cells, and 48 h post-transfection, the cells expressing GFP were sorted as one cell per well in a 96-well plate. These cells were grown and analyzed for PTB knockout using western blotting. The gRNA sequence is GCTCCCCATCGACGTCACGG.

Upon western blotting, we found that we could only obtain a partial knockdown of PTB; therefore, the cell line is described as PTB knockdown cell line.

### MTT assay

Proliferation was measured using 3-(4,5-dimethylthiazol-2-yl)-2,5-diphenyltetrazolium bromide (MTT). MTT was added to the cells at a final concentration of 0.5 mg/mL at the desired time points. After 3–4 h, media was removed, cells were treated with 100 µL DMSO, and the absorbance was measured at 560 nm.

### IP-RT

HEK-293T-ACE2 cells were infected with the SARS-CoV-2 virus at an MOI of 0.1 and after completion of the experiment, 1% formaldehyde was added to the media and incubated at 4°C for 10 min. Thereafter, the reaction was quenched with 0.1 M glycine incubated at 4°C for 10 min. The cells were then washed with PBS and lysed in polysome lysis buffer (100 mM KCl, 10 mM HEPES pH 7.0, 5 mM MgCl_2_, 0.5% NP-40, 1 mM DTT, 100 U/mL RNasin). The supernatant obtained was precleared with Protein G Sepharose beads. In parallel, the Protein G Sepharose beads were incubated with 1 µg of HuR/PTB antibody overnight at 4°C and added to the precleared lysates, followed by overnight incubation with continuous mixing on a rotator device at 4°C. The beads were then washed three times with polysome lysis buffer. SDS sample buffer was added to the 20% of beads and boiled to release the immunoprecipitated protein, and the supernatant was electrophoresed on SDS-12% PAGE. The RNA was isolated from the remaining 80% of beads. The beads were incubated with 0.1% SDS and 30 µg proteinase K at 50°C for 30 min, and RNA was isolated after addition of three times volume of Tri Reagent.

### RNA isolation followed by real-time PCR

The total RNA from ACE2-HEK-293T cells was isolated using TRI Reagent (Sigma) as per manufacturer’s protocol. cDNA was synthesized using the following primers: CoV-2 Fwd: 5′ TGTCGTTGACAGGACACGAG 3′, CoV-2 Rev: 5′ TTACCTTTCGGTCACACCCG 3′, GAPDH Fwd: 5′ CAGCCTCAAGATCATCAGCAAT 3′, GAPDH Rev: 5′ GGTCATGAGTCCTTCCACGA 3′. For quantification of viral positive strand, CoV-2 Rev and GAPDH Rev primers were used for cDNA synthesis. For quantification of viral negative strand, CoV-2 Fwd and GAPDH Rev primers were used for cDNA synthesis. Moloney murine leukemia virus reverse transcriptase was used for cDNA synthesis at 42°C for 1 h using 600 ng of total RNA. Quantitative RT-PCR (qRT-PCR) was done using DyNAmo HS SYBR Green qPCR kit (ThermoScientific). qRT-PCR was done using 2 µL of cDNA in a 10 µL reaction mixture according to the manufacturer’s instructions for 40 cycles. The comparative threshold cycle (CT) method was used to calculate fold change in SARS-CoV-2 RNA levels (2^-∆∆CT^) and normalized to GAPDH. For ex vivo IP-RT assay using HuR, ΔCt was calculated using respective IgG pulldown samples from mock and SARS-CoV-2 infection, and ΔΔCt was calculated by normalizing the values to mock-infected samples. Fold change was calculated as 2^(-ΔΔCt)^.

### Western blotting

Protein concentrations of the extracts were assayed by Bradford reagent (Bio-Rad), and equal amounts of cell extracts were separated by SDS-10% PAGE and transferred onto a nitrocellulose membrane (Sigma). Samples were then analyzed by western blot using the desired antibodies, anti-SARS-CoV-2 N protein (40143-MM05, Sino Biological), anti-HuR antibody (3A2, Santa Cruz), anti-Myc tag antibody (ab9106), anti-SARS-CoV-2 Spike antibody (GTX632604, Genetex), anti-SARS-CoV-2 Nsp5 antibody (GTX135470, Genetex), anti-GAPDH antibody (sc47724, Santa Cruz), anti-LaminB1 antibody (sc374015, Santa Cruz), anti-PTB antibody (MABE986, Sigma-Aldrich), or anti-ACE2 antibody (ab15348) followed by the respective secondary antibodies (horseradish peroxidase-conjugated anti-mouse or anti-rabbit IgG; Sigma). Mouse-monoclonal anti-β-actin-peroxidase antibody (A3854, Sigma) was used as a control for equal loading of total cell extracts. Antibody complexes were detected using the Immobilon Western systems (Millipore).

### Sucrose gradient polysome fractionation

After completion of the experiment, cells were treated with 100 µg/mL cycloheximide for 10 min at 37°C. Thereafter, media was removed and cells washed first with ice-cold PBS containing 100 µg/mL cycloheximide and then with 1× hypotonic buffer (5 mM TRIS-HCl, pH 7.5, 5 mM MgCl_2_ and 1.5 mM KCl) containing 100 µg/mL cycloheximide. Cells were scraped in 350 µL ice-cold lysis buffer (5 mM TRIS-HCl, pH 7.5, 5 mM MgCl_2_, 1.5 mM KCl, 100 µg/mL cycloheximide, 1 mM DTT, 200 U/mL RNase from Promega, 0.5% sodium deoxycholate, 0.5% Triton X-100, 200 µg tRNA, and 1× protease inhibitor cocktail) and incubated for 15 min on ice. The KCl concentration in the lysate was adjusted to 150 mM. The lysate was spun for 8 min at 3,000 × *g* at 4°C, and supernatant was collected. Five hundred micrograms of the lysate was loaded on the top of 15%–50% sucrose gradient containing 100 µg/mL cycloheximide, and the gradients were centrifuged at 36,000 rpm for 2 h at 4°C in SW41 rotor (Beckman). Density Gradient Fractionation System (ISCO) was used to fractionate the gradients at a flow rate of 0.3 mm/s with the UV detector sensitivity set at 1.0. Monosome and polysome fractions were pooled to isolate RNA using Trizol (Sigma). SARS-CoV-2 RNA level was quantified using real-time PCR.

#### Primers

Primers used for PCR were as follows: genomic 5′-UTR forward, TGTCGTTGACAGGACACGAG; genomic 5′-UTR reverse, TTACCTTTCGGTCACACCCG; N sgmRNA LSf forward, CGATCTCTTGTAGATCTGTTC; N sgmRNA reverse, AGCGGTGAACCAAGACGCA; S sgmRNA LSf forward, CCAACTTTCGATCTCTTGTAG; S sgmRNA reverse, AGAACAAGTCCTGAGTTGAATG; IL-6 forward, GGTACATCCTCGACGGCATCT; IL-6 reverse, GTGCCTCTTTGCTGCTTTCAC. Primers used for HuR gene PCR were as follows: forward primer, TACTTGCTCTTTTTCTCTTGGC; reverse primer, CACAGCCATCGTTTCAAGGC. Antisense oligonucleotides were as follows: Ctrl ASO, CGTTAGATTACCGCG; ASO5, CAACACGGACGAAACC.

### Software

All emerging lineages of SARS-CoV-2 were downloaded from Global Initiative on Sharing All Influenza Data (https://www.gisaid.org/). For multiple sequence alignment, the Clustal Omega online tool (https://www.ebi.ac.uk/Tools/msa/clustalo/) was used. To predict the secondary structure, the RNAstructure online tool was used (https://rna.urmc.rochester.edu/RNAstructureWeb/Servers/). RNA-binding protein interactions to SARS-CoV-2 5′-UTR were predicted with RBPDB (http://rbpdb.ccbr.utoronto.ca/). Binding regions on the RNA sequence were predicted with Cat-Rapid fragment (http://service.tartaglialab.com/page/catrapid_group).

Data were plotted using GraphPad Prism.

## Data Availability

The authors confirm that the data supporting the findings of this study are available within the article and its supplemental material.

## References

[1] V’kovski P, Kratzel A, Steiner S, Stalder H, Thiel V. 2021. Coronavirus biology and replication: implications for SARS-CoV-2. Nat Rev Microbiol 19:155–170. doi:10.1038/s41579-020-00468-6 33116300 PMC7592455

[2] Embarc-Buh A, Francisco-Velilla R, Martinez-Salas E. 2021. RNA-binding proteins at the host-pathogen interface targeting viral regulatory Elements. Viruses 13:952. doi:10.3390/v13060952 34064059 PMC8224014

[3] Lisy S, Rothamel K, Ascano M. 2021. RNA binding proteins as pioneer determinants of infection: protective, proviral, or both? Viruses 13:2172. doi:10.3390/v13112172 34834978 PMC8625426

[4] Kamel W, Noerenberg M, Cerikan B, Chen H, Järvelin AI, Kammoun M, Lee JY, Shuai N, Garcia-Moreno M, Andrejeva A, Deery MJ, Johnson N, Neufeldt CJ, Cortese M, Knight ML, Lilley KS, Martinez J, Davis I, Bartenschlager R, Mohammed S, Castello A. 2021. Global analysis of protein-RNA interactions in SARS-CoV-2-infected cells reveals key regulators of infection. Mol Cell 81:2851–2867. doi:10.1016/j.molcel.2021.05.023 34118193 PMC8142890

[5] Gordon DE, Jang GM, Bouhaddou M, Xu J, Obernier K, White KM, O’Meara MJ, Rezelj VV, Guo JZ, Swaney DL, et al.. 2020. A SARS-CoV-2 protein interaction map reveals targets for drug repurposing. Nature 583:459–468. doi:10.1038/s41586-020-2286-9 32353859 PMC7431030

[6] Mukherjee N, Corcoran DL, Nusbaum JD, Reid DW, Georgiev S, Hafner M, Ascano M Jr, Tuschl T, Ohler U, Keene JD. 2011. Integrative regulatory mapping indicates that the RNA-binding protein HuR couples pre-mRNA processing and mRNA stability. Mol Cell 43:327–339. doi:10.1016/j.molcel.2011.06.007 21723170 PMC3220597

[7] Lebedeva S, Jens M, Theil K, Schwanhäusser B, Selbach M, Landthaler M, Rajewsky N. 2011. Transcriptome-wide analysis of regulatory interactions of the RNA-binding protein HuR. Mol Cell 43:340–352. doi:10.1016/j.molcel.2011.06.008 21723171

[8] Grammatikakis I, Abdelmohsen K, Gorospe M. 2017. Posttranslational control of HuR function. Wiley Interdiscip Rev RNA 8:10. doi:10.1002/wrna.1372 PMC5607777 27307117

[9] Shwetha S, Kumar A, Mullick R, Vasudevan D, Mukherjee N, Das S. 2015. HuR displaces polypyrimidine tract binding protein to facilitate la binding to the 3’ untranslated region and enhances hepatitis C virus replication. J Virol 89:11356–11371. doi:10.1128/JVI.01714-15 26339049 PMC4645635

[10] Raheja H, George B, Tripathi SK, Saha S, Maiti TK, Das S. 2023. Hepatitis C virus non-structural proteins modulate cellular kinases for increased cytoplasmic abundance of host factor HuR and facilitate viral replication. PLoS Pathog 19:e1011552. doi:10.1371/journal.ppat.1011552 37540723 PMC10431626

[11] George B, Dave P, Rani P, Behera P, Das S. 2021. Cellular protein HuR regulates the switching of genomic RNA templates for differential functions during the coxsackievirus B3 life cycle. J Virol 95:e0091521. doi:10.1128/JVI.00915-21 34406862 PMC8513481

[12] Jehung JP, Kitamura T, Yanagawa-Matsuda A, Kuroshima T, Towfik A, Yasuda M, Sano H, Kitagawa Y, Minowa K, Shindoh M, Higashino F. 2018. Adenovirus infection induces HuR relocalization to facilitate virus replication. Biochem Biophys Res Commun 495:1795–1800. doi:10.1016/j.bbrc.2017.12.036 29225167

[13] Sokoloski KJ, Dickson AM, Chaskey EL, Garneau NL, Wilusz CJ, Wilusz J. 2010. Sindbis virus usurps the cellular HuR protein to stabilize its transcripts and promote productive infections in mammalian and mosquito cells. Cell Host & Microbe 8:196–207. doi:10.1016/j.chom.2010.07.003 20709296 PMC2929003

[14] Lemay J, Maidou-Peindara P, Bader T, Ennifar E, Rain J-C, Benarous R, Liu LX. 2008. HuR interacts with human immunodeficiency virus type 1 reverse transcriptase, and modulates reverse transcription in infected cells. Retrovirology (Auckl) 5:47. doi:10.1186/1742-4690-5-47 PMC2441633 18544151

[15] Eskandarzade N, Ghorbani A, Samarfard S, Diaz J, Guzzi PH, Fariborzi N, Tahmasebi A, Izadpanah K. 2022. Network for network concept offers new insights into host- SARS-CoV-2 protein interactions and potential novel targets for developing antiviral drugs. Comput Biol Med 146:105575. doi:10.1016/j.compbiomed.2022.105575 35533462 PMC9055686

[16] Miao Z, Tidu A, Eriani G, Martin F. 2021. Secondary structure of the SARS-CoV-2 5’-UTR. RNA Biol 18:447–456. doi:10.1080/15476286.2020.1814556 32965173 PMC7544965

[17] Cook KB, Kazan H, Zuberi K, Morris Q, Hughes TR. 2011. RBPDB: a database of RNA-binding specificities. Nucleic Acids Res 39:D301–8. doi:10.1093/nar/gkq1069 21036867 PMC3013675

[18] Ouhara K, Munenaga S, Kajiya M, Takeda K, Matsuda S, Sato Y, Hamamoto Y, Iwata T, Yamasaki S, Akutagawa K, Mizuno N, Fujita T, Sugiyama E, Kurihara H. 2018. The induced RNA-binding protein, HuR, targets 3’-UTR region of IL-6 mRNA and enhances its stabilization in periodontitis. Clin Exp Immunol 192:325–336. doi:10.1111/cei.13110 29393507 PMC5980314

[19] Alexandersen S, Chamings A, Bhatta TR. 2020. SARS-CoV-2 genomic and subgenomic RNAs in diagnostic samples are not an indicator of active replication. Nat Commun 11:6059. doi:10.1038/s41467-020-19883-7 33247099 PMC7695715

[20] Khan D, Terenzi F, Liu G, Ghosh PK, Ye F, Nguyen K, China A, Ramachandiran I, Chakraborty S, Stefan J, Khan K, Vasu K, Dong F, Willard B, Karn J, Gack MU, Fox PL. 2023. A viral pan-end RNA element and host complex define a SARS-CoV-2 regulon. Nat Commun 14:3385. doi:10.1038/s41467-023-39091-3 37296097 PMC10250186

[21] Long S. 2021. SARS-CoV-2 subgenomic RNAs: characterization, utility, and perspectives. Viruses 13:1923. doi:10.3390/v13101923 34696353 PMC8539008

[22] Zhu C, Lee JY, Woo JZ, Xu L, Wrynla XH, Yamashiro LH, Ji F, Biering SB, Van Dis E, Gonzalez F, Fox D, Wehri E, Rustagi A, Pinsky BA, Schaletzky J, Blish CA, Chiu C, Harris E, Sadreyev RI, Stanley S, Kauppinen S, Rouskin S, Näär AM. 2022. An intranasal ASO therapeutic targeting SARS-CoV-2. Nat Commun 13:4503. doi:10.1038/s41467-022-32216-0 35922434 PMC9349213

[23] Hart WS, Miller E, Andrews NJ, Waight P, Maini PK, Funk S, Thompson RN. 2022. Generation time of the alpha and delta SARS-CoV-2 variants: an epidemiological analysis. Lancet Infect Dis 22:603–610. doi:10.1016/S1473-3099(22)00001-9 35176230 PMC8843191

[24] Tidu A, Janvier A, Schaeffer L, Sosnowski P, Kuhn L, Hammann P, Westhof E, Eriani G, Martin F. 2020. The viral protein NSP1 acts as a ribosome gatekeeper for shutting down host translation and fostering SARS-CoV-2 translation. Molecular Biology. doi:10.1101/2020.10.14.339515 PMC7901841 33268501

[25] Schubert K, Karousis ED, Jomaa A, Scaiola A, Echeverria B, Gurzeler L-A, Leibundgut M, Thiel V, Mühlemann O, Ban N. 2020. SARS-CoV-2 Nsp1 binds the ribosomal mRNA channel to inhibit translation. Nat Struct Mol Biol 27:959–966. doi:10.1038/s41594-020-0511-8 32908316

[26] Kehrer T, Cupic A, Ye C, Yildiz S, Bouhhadou M, Crossland NA, Barrall E, Cohen P, Tseng A, Çağatay T, et al.. 2022. Impact of SARS-CoV-2 ORF6 and its variant polymorphisms on host responses and viral pathogenesis. bioRxiv:2022.10.18.512708. doi:10.1101/2022.10.18.512708 PMC10750313 37738983

[27] Schmidt N, Ganskih S, Wei Y, Gabel A, Zielinski S, Keshishian H, Lareau CA, Zimmermann L, Makroczyova J, Pearce C, et al.. 2023. SND1 binds SARS-CoV-2 negative-sense RNA and promotes viral RNA synthesis through NSP9. Cell 186:4834–4850. doi:10.1016/j.cell.2023.09.002 37794589 PMC10617981

[28] Srikantan S, Gorospe M. 2012. HuR function in disease. Front Biosci 17:189. doi:10.2741/3921 PMC4540328 22201738

[29] Banerjee A, El-Sayes N, Budylowski P, Jacob RA, Richard D, Maan H, Aguiar JA, Demian WL, Baid K, D’Agostino MR, et al.. 2021. Experimental and natural evidence of SARS-CoV-2-infection-induced activation of type I interferon responses. iScience 24:102477. doi:10.1016/j.isci.2021.102477 33937724 PMC8074517

[30] Shwetha S, Sharma G, Raheja H, Goel A, Aggarwal R, Das S. 2018. Interaction of miR-125b-5p with Human antigen R mRNA: mechanism of controlling HCV replication. Virus Res 258:1–8. doi:10.1016/j.virusres.2018.09.006 30253192

[31] Raguraman R, Shanmugarama S, Mehta M, Elle Peterson J, Zhao YD, Munshi A, Ramesh R. 2022. Drug delivery approaches for HuR-targeted therapy for lung cancer. Adv Drug Deliv Rev 180:114068. doi:10.1016/j.addr.2021.114068 34822926 PMC8724414

[32] Majumder M, Chakraborty P, Mohan S, Mehrotra S, Palanisamy V. 2022. HuR as a molecular target for cancer therapeutics and immune-related disorders. Adv Drug Deliv Rev 188:114442. doi:10.1016/j.addr.2022.114442 35817212 PMC10276344

[33] Wiedemar N, Hauser DA, Mäser P. 2020. 100 years of suramin. Antimicrob Agents Chemother 64:e01168-19. doi:10.1128/AAC.01168-19 PMC7038244 31844000

[34] Salgado-Benvindo C, Thaler M, Tas A, Ogando NS, Bredenbeek PJ, Ninaber DK, Wang Y, Hiemstra PS, Snijder EJ, van Hemert MJ. 2020. Suramin inhibits SARS-CoV-2 infection in cell culture by interfering with early steps of the replication cycle. Antimicrob Agents Chemother 64:e00900-20. doi:10.1128/AAC.00900-20 32513797 PMC7526844

[35] Yin W, Luan X, Li Z, Zhou Z, Wang Q, Gao M, Wang X, Zhou F, Shi J, You E, Liu M, Wang Q, Jiang Y, Jiang H, Xiao G, Zhang L, Yu X, Zhang S, Eric Xu H. 2021. Structural basis for inhibition of the SARS-CoV-2 RNA polymerase by suramin. Nat Struct Mol Biol 28:319–325. doi:10.1038/s41594-021-00570-0 33674802

[36] Kwon PS, Xu S, Oh H, Kwon SJ, Rodrigues AL, Feroz M, Fraser K, He P, Zhang F, Hong JJ, Linhardt RJ, Dordick JS. 2023. Suramin binds and inhibits infection of SARS-CoV-2 through both spike protein-heparan sulfate and ACE2 receptor interactions. Commun Biol 6:387. doi:10.1038/s42003-023-04789-z 37031303 PMC10082822

[37] Boniardi I, Corona A, Basquin J, Basquin C, Milia J, Nagy I, Tramontano E, Zinzula L. 2023. Suramin inhibits SARS-CoV-2 nucleocapsid phosphoprotein genome packaging function. Virus Res 336:199221. doi:10.1016/j.virusres.2023.199221 37704176 PMC10514558

[38] Case JB, Bailey AL, Kim AS, Chen RE, Diamond MS. 2020. Growth, detection, quantification, and inactivation of SARS-CoV-2. Virology (Auckl) 548:39–48. doi:10.1016/j.virol.2020.05.015 PMC7293183 32838945

